# Supplementary material for: Human embryonic lung epithelial tips are multipotent progenitors that can be expanded in vitro as long-term self-renewing organoids
Source: eLife. 2017 Jun 30;6:e26575. doi: 10.7554/eLife.26575 (PMC5555721; doi:10.7554/eLife.26575)
Supplement: Supplementary file 2. — DOI: http://dx.doi.org/10.7554/eLife.26575.046 [file elife-26575-supp2.docx]

**Supplemental Table 2** Transcription Factors with mouse orthologues upregulated >2-fold in human tips or stalks

**Tips**

| **Gene Symbol** | **Known tip expression?** | **Reference** |
| --- | --- | --- |
| *ARID3B* | No |  |
| *CEBPA* | No |  |
| *E2F1* | Yes | Herriges, J.C., Yi, L., Hines, E.A., Harvey, J.F., Xu, G., Gray, P.A., Ma, Q., Sun, X., 2012. Genome-scale study of transcription factor expression in the branching mouse lung. Dev Dyn 241, 1432-1453. |
| *E2F8* | No |  |
| *ELF5* | Yes | Metzger, D.E., Xu, Y., Shannon, J.M., 2007. Elf5 is an epithelium-specific, fibroblast growth factor-sensitive transcription factor in the embryonic lung. Dev Dyn 236, 1175-1192. |
| *ETS2* | Yes | Herriges, J.C., Yi, L., Hines, E.A., Harvey, J.F., Xu, G., Gray, P.A., Ma, Q., Sun, X., 2012. Genome-scale study of transcription factor expression in the branching mouse lung. Dev Dyn 241, 1432-1453. |
| *ETV1* | No |  |
| *ETV4* | Yes | Liu, Y., Jiang, H., Crawford, H.C., Hogan, B.L., 2003. Role for ETS domain transcription factors Pea3/Erm in mouse lung development. Dev Biol 261, 10-24. |
| *ETV5* | Yes | Liu, Y., Hogan, B.L., 2002. Differential gene expression in the distal tip endoderm of the embryonic mouse lung. Gene Expr Patterns 2, 229-233. |
| *GATA6* | Yes | Keijzer, R., van Tuyl, M., Meijers, C., Post, M., Tibboel, D., Grosveld, F., Koutsourakis, M., 2001. The transcription factor GATA6 is essential for branching morphogenesis and epithelial cell differentiation during fetal pulmonary development. Development 128, 503-511.  Morrisey, E.E., Ip, H.S., Lu, M.M., Parmacek, M.S., 1996. GATA-6: a zinc finger transcription factor that is expressed in multiple cell lineages derived from lateral mesoderm. Dev Biol 177, 309-322. |
| *HMGA1* | No |  |
| *HMGA2* | Yes | Singh, I., Mehta, A., Contreras, A., Boettger, T., Carraro, G., Wheeler, M., Cabrera-Fuentes, H.A., Bellusci, S., Seeger, W., Braun, T., Barreto, G., 2014. Hmga2 is required for canonical WNT signaling during lung development. BMC biology 12, 21. |
| *HNF1B* | Yes | Herriges, J.C., Yi, L., Hines, E.A., Harvey, J.F., Xu, G., Gray, P.A., Ma, Q., Sun, X., 2012. Genome-scale study of transcription factor expression in the branching mouse lung. Dev Dyn 241, 1432-1453. |
| *ID1* | No |  |
| *ID2* | Yes | Liu, Y., Hogan, B.L., 2002. Differential gene expression in the distal tip endoderm of the embryonic mouse lung. Gene Expr Patterns 2, 229-233. |
| *ID3* | No |  |
| *IRX1* | Yes | Becker, M.B., Zulch, A., Bosse, A., Gruss, P., 2001. Irx1 and Irx2 expression in early lung development. Mech Dev 106, 155-158. |
| *IRX5* | Yes | van Tuyl, M., Liu, J., Groenman, F., Ridsdale, R., Han, R.N., Venkatesh, V., Tibboel, D., Post, M., 2006. Iroquois genes influence proximo-distal morphogenesis during rat lung development. Am J Physiol Lung Cell Mol Physiol 290, L777-L789. |
| *IRX6* | Yes | Herriges, J.C., Yi, L., Hines, E.A., Harvey, J.F., Xu, G., Gray, P.A., Ma, Q., Sun, X., 2012. Genome-scale study of transcription factor expression in the branching mouse lung. Dev Dyn 241, 1432-1453. |
| *KLF15* | Yes | Diez-Roux, G., Banfi, S., Sultan, M., et al., 2011. A high-resolution anatomical atlas of the transcriptome in the mouse embryo. PLoS Biol 9, e1000582. |
| *LEF1* | Yes | Okubo, T., Hogan, B.L., 2004. Hyperactive Wnt signaling changes the developmental potential of embryonic lung endoderm. J Biol 3, 11. |
| *MEIS2* | No |  |
| *MSX2* | No |  |
| *MYBL2* | Yes | Diez-Roux, G., Banfi, S., Sultan, M., et al., 2011. A high-resolution anatomical atlas of the transcriptome in the mouse embryo. PLoS Biol 9, e1000582. |
| *MYCN* | Yes | Lu, J., Qian, J., Izvolsky, K.I., Cardoso, W.V., 2004. Global analysis of genes differentially expressed in branching and non-branching regions of the mouse embryonic lung. Dev Biol 273, 418-435. |
| *NFE2* | Yes | Diez-Roux, G., Banfi, S., Sultan, M., et al., 2011. A high-resolution anatomical atlas of the transcriptome in the mouse embryo. PLoS Biol 9, e1000582. |
| *NFE2L3* | No |  |
| *NPAS2* | No |  |
| *RFX6* | No |  |
| *SALL4* | No |  |
| *SOX9* | Yes | Liu, Y., Jiang, H., Crawford, H.C., Hogan, B.L., 2003. Role for ETS domain transcription factors Pea3/Erm in mouse lung development. Dev Biol 261, 10-24. |
| *SP5* | Yes | Diez-Roux, G., Banfi, S., Sultan, M., et al., 2011. A high-resolution anatomical atlas of the transcriptome in the mouse embryo. PLoS Biol 9, e1000582. |
| *TCF7* | Yes | Tebar, M., Destree, O., de Vree, W.J., Ten Have-Opbroek, A.A., 2001. Expression of Tcf/Lef and sFrp and localization of beta-catenin in the developing mouse lung. Mech Dev 109, 437-440. |
| *TGIF1* | Yes | Diez-Roux, G., Banfi, S., Sultan, M., et al., 2011. A high-resolution anatomical atlas of the transcriptome in the mouse embryo. PLoS Biol 9, e1000582. |
| *ZNF319* | No |  |
| *ZNF492* | No |  |
| *ZNF98* | No |  |

**Stalks**

| **Gene Symbol** | **Mesenchymal expression?** | **Reference** |
| --- | --- | --- |
| *ASCL1* | No |  |
| *BACH2* | No |  |
| *BCL6* | No |  |
| *BNC1* | No |  |
| *BNC2* | No |  |
| *CASZ1* | Yes | Charpentier, M.S., Christine, K.S., Amin, N.M., Dorr, K.M., Kushner, E.J., Bautch, V.L., Taylor, J.M., Conlon, F.L., 2013. CASZ1 promotes vascular assembly and morphogenesis through the direct regulation of an EGFL7/RhoA-mediated pathway. Dev Cell 25, 132-143. |
| *CBX4* | No |  |
| *CBX6* | No |  |
| *CREB3L1* | No |  |
| *CREB5* | No |  |
| *DLX1* | No |  |
| *EHF* | No |  |
| *ETS1* | Yes | Herriges, J.C., Yi, L., Hines, E.A., Harvey, J.F., Xu, G., Gray, P.A., Ma, Q., Sun, X., 2012. Genome-scale study of transcription factor expression in the branching mouse lung. Dev Dyn 241, 1432-1453. |
| *ETV3* | No |  |
| *FHL1* | Yes | Chu, P.H., Ruiz-Lozano, P., Zhou, Q., Cai, C., Chen, J., 2000. Expression patterns of FHL/SLIM family members suggest important functional roles in skeletal muscle and cardiovascular system. Mech Dev 95, 259-265. |
| *FHL2* | Yes | Chu, P.H., Ruiz-Lozano, P., Zhou, Q., Cai, C., Chen, J., 2000. Expression patterns of FHL/SLIM family members suggest important functional roles in skeletal muscle and cardiovascular system. Mech Dev 95, 259-265. |
| *FHL3* | No |  |
| *FOSL2* | No |  |
| *FOXF1* | Yes | Mahlapuu, M., Enerback, S., Carlsson, P., 2001. Haploinsufficiency of the forkhead gene Foxf1, a target for sonic hedgehog signaling, causes lung and foregut malformations. Development 128, 2397-2406. |
| *FOXF2* | Yes | Aitola, M., Carlsson, P., Mahlapuu, M., Enerback, S., Pelto-Huikko, M., 2000. Forkhead transcription factor FoxF2 is expressed in mesodermal tissues involved in epithelio-mesenchymal interactions. Dev Dyn 218, 136-149. |
| *FOXO1* | No |  |
| *GATA5* | No |  |
| *GLIS3* | No |  |
| *GRHL1* | No |  |
| *HAND2* | No |  |
| *HES6* | No |  |
| *HEYL* | Yes | Leimeister, C., Schumacher, N., Steidl, C., Gessler, M., 2000. Analysis of HeyL expression in wild-type and Notch pathway mutant mouse embryos. Mech Dev 98, 175-178. |
| *HIC1* | No |  |
| *HIF3A* | No |  |
| *HIVEP3* | No |  |
| *HOXA2* | Yes | Boucherat, O., Montaron, S., Berube-Simard, F.A., Aubin, J., Philippidou, P., Wellik, D.M., Dasen, J.S., Jeannotte, L., 2013. Partial functional redundancy between Hoxa5 and Hoxb5 paralog genes during lung morphogenesis. Am J Physiol Lung Cell Mol Physiol 304, L817-830. |
| *HOXA3* | Yes | Boucherat, O., Montaron, S., Berube-Simard, F.A., Aubin, J., Philippidou, P., Wellik, D.M., Dasen, J.S., Jeannotte, L., 2013. Partial functional redundancy between Hoxa5 and Hoxb5 paralog genes during lung morphogenesis. Am J Physiol Lung Cell Mol Physiol 304, L817-830. |
| *HOXA4* | Yes | Boucherat, O., Montaron, S., Berube-Simard, F.A., Aubin, J., Philippidou, P., Wellik, D.M., Dasen, J.S., Jeannotte, L., 2013. Partial functional redundancy between Hoxa5 and Hoxb5 paralog genes during lung morphogenesis. Am J Physiol Lung Cell Mol Physiol 304, L817-830. |
| *HOXA5* | Yes | Boucherat, O., Montaron, S., Berube-Simard, F.A., Aubin, J., Philippidou, P., Wellik, D.M., Dasen, J.S., Jeannotte, L., 2013. Partial functional redundancy between Hoxa5 and Hoxb5 paralog genes during lung morphogenesis. Am J Physiol Lung Cell Mol Physiol 304, L817-830. |
| *HOXB2* | Yes | Boucherat, O., Montaron, S., Berube-Simard, F.A., Aubin, J., Philippidou, P., Wellik, D.M., Dasen, J.S., Jeannotte, L., 2013. Partial functional redundancy between Hoxa5 and Hoxb5 paralog genes during lung morphogenesis. Am J Physiol Lung Cell Mol Physiol 304, L817-830. |
| *HOXB4* | Yes | Boucherat, O., Montaron, S., Berube-Simard, F.A., Aubin, J., Philippidou, P., Wellik, D.M., Dasen, J.S., Jeannotte, L., 2013. Partial functional redundancy between Hoxa5 and Hoxb5 paralog genes during lung morphogenesis. Am J Physiol Lung Cell Mol Physiol 304, L817-830. |
| *HOXB5* | Yes | Boucherat, O., Montaron, S., Berube-Simard, F.A., Aubin, J., Philippidou, P., Wellik, D.M., Dasen, J.S., Jeannotte, L., 2013. Partial functional redundancy between Hoxa5 and Hoxb5 paralog genes during lung morphogenesis. Am J Physiol Lung Cell Mol Physiol 304, L817-830. |
| *HOXC4* | Yes | Boucherat, O., Montaron, S., Berube-Simard, F.A., Aubin, J., Philippidou, P., Wellik, D.M., Dasen, J.S., Jeannotte, L., 2013. Partial functional redundancy between Hoxa5 and Hoxb5 paralog genes during lung morphogenesis. Am J Physiol Lung Cell Mol Physiol 304, L817-830. |
| *INSM1* | No |  |
| *KLF11* | No |  |
| *KLF4* | No |  |
| *KLF5* | No |  |
| *KLF6* | No |  |
| *LDB2* | No |  |
| *LHX6* | Yes | Diez-Roux, G., Banfi, S., Sultan, M., et al., 2011. A high-resolution anatomical atlas of the transcriptome in the mouse embryo. PLoS Biol 9, e1000582. |
| *LMO3* | No |  |
| *LMO4* | No |  |
| *MAF* | No |  |
| *MAFB* | No |  |
| *MECOM* | No |  |
| *MEF2C* | Yes | Diez-Roux, G., Banfi, S., Sultan, M., et al., 2011. A high-resolution anatomical atlas of the transcriptome in the mouse embryo. PLoS Biol 9, e1000582. |
| *MEIS1* | Yes | Diez-Roux, G., Banfi, S., Sultan, M., et al., 2011. A high-resolution anatomical atlas of the transcriptome in the mouse embryo. PLoS Biol 9, e1000582. |
| *MEIS3* | No |  |
| *MEOX2* | Yes | Diez-Roux, G., Banfi, S., Sultan, M., et al., 2011. A high-resolution anatomical atlas of the transcriptome in the mouse embryo. PLoS Biol 9, e1000582. |
| *MITF* | No |  |
| *MYC* | No |  |
| *MYOCD* | Yes | Espinoza-Lewis, R.A., Wang, D.Z., 2014. Generation of a Cre knock-in into the Myocardin locus to mark early cardiac and smooth muscle cell lineages. Genesis 52, 879-887. |
| *MYT1* | No |  |
| *NFATC1* | No |  |
| *NFATC4* | No |  |
| *NFIA* | Yes | Chaudhry, A.Z., Lyons, G.E., Gronostajski, R.M., 1997. Expression patterns of the four nuclear factor I genes during mouse embryogenesis indicate a potential role in development. Dev Dyn 208, 313-325. |
| *NFIB* | Yes | Chaudhry, A.Z., Lyons, G.E., Gronostajski, R.M., 1997. Expression patterns of the four nuclear factor I genes during mouse embryogenesis indicate a potential role in development. Dev Dyn 208, 313-325. |
| *NFIC* | Yes | Chaudhry, A.Z., Lyons, G.E., Gronostajski, R.M., 1997. Expression patterns of the four nuclear factor I genes during mouse embryogenesis indicate a potential role in development. Dev Dyn 208, 313-325. |
| *NFIX* | Yes | Chaudhry, A.Z., Lyons, G.E., Gronostajski, R.M., 1997. Expression patterns of the four nuclear factor I genes during mouse embryogenesis indicate a potential role in development. Dev Dyn 208, 313-325. |
| *NPAS3* | No |  |
| *NR2F1* | No |  |
| *NR3C1* | Yes | Laresgoiti, U., Nikolic, M.Z., Rao, C., Brady, J.L., Richardson, R.V., Batchen, E.J., Chapman, K.E., Rawlins, E.L., 2016. Lung epithelial tip progenitors integrate glucocorticoid- and STAT3-mediated signals to control progeny fate. Development 143, 3686-3699. |
| *OSR1* | No |  |
| *PBX3* | Yes | Di Giacomo, G., Koss, M., Capellini, T.D., Brendolan, A., Popperl, H., Selleri, L., 2006. Spatio-temporal expression of Pbx3 during mouse organogenesis. Gene Expr Patterns 6, 747-757. |
| *PHOX2B* | No |  |
| *PLAG1* | No |  |
| *PLAGL1* | No |  |
| *POU2F2* | No |  |
| *PPARGC1A* | No |  |
| *PRDM6* | Yes | Davis, C.A., Haberland, M., Arnold, M.A., Sutherland, L.B., McDonald, O.G., Richardson, J.A., Childs, G., Harris, S., Owens, G.K., Olson, E.N., 2006. PRISM/PRDM6, a transcriptional repressor that promotes the proliferative gene program in smooth muscle cells. Mol Cell Biol 26, 2626-2636. |
| *PROX1* | Yes | Gordon, E.J., Gale, N.W., Harvey, N.L., 2008. Expression of the hyaluronan receptor LYVE-1 is not restricted to the lymphatic vasculature; LYVE-1 is also expressed on embryonic blood vessels. Dev Dyn 237, 1901-1909. |
| *RORA* | No |  |
| *RUNX1* | No |  |
| *RUNX1T1* | No |  |
| *SCAND1* | No |  |
| *SDPR* | Yes | Hansen, C.G., Shvets, E., Howard, G., Riento, K., Nichols, B.J., 2013. Deletion of cavin genes reveals tissue-specific mechanisms for morphogenesis of endothelial caveolae. Nat Commun 4, 1831. |
| *SETBP1* | No |  |
| *SNAI2* | No |  |
| *SOX2* | No |  |
| *SOX21* | No |  |
| *SP6* | No |  |
| *TBX1* | Yes | Arora, R., Metzger, R.J., Papaioannou, V.E., 2012. Multiple roles and interactions of Tbx4 and Tbx5 in development of the respiratory system. PLoS genetics 8, e1002866. |
| *TBX2* | Yes | Arora, R., Metzger, R.J., Papaioannou, V.E., 2012. Multiple roles and interactions of Tbx4 and Tbx5 in development of the respiratory system. PLoS genetics 8, e1002866. |
| *TBX3* | Yes | Arora, R., Metzger, R.J., Papaioannou, V.E., 2012. Multiple roles and interactions of Tbx4 and Tbx5 in development of the respiratory system. PLoS genetics 8, e1002866. |
| *TBX4* | Yes | Arora, R., Metzger, R.J., Papaioannou, V.E., 2012. Multiple roles and interactions of Tbx4 and Tbx5 in development of the respiratory system. PLoS genetics 8, e1002866. |
| *TBX5* | Yes | Arora, R., Metzger, R.J., Papaioannou, V.E., 2012. Multiple roles and interactions of Tbx4 and Tbx5 in development of the respiratory system. PLoS genetics 8, e1002866. |
| *TCF12* | No |  |
| *TCF21* | Yes | Quaggin, S.E., Schwartz, L., Cui, S., Igarashi, P., Deimling, J., Post, M., Rossant, J., 1999. The basic-helix-loop-helix protein pod1 is critically important for kidney and lung organogenesis. Development 126, 5771-5783. |
| *TCF4* | No |  |
| *TCF7L2* | No |  |
| *TFAP2C* | No |  |
| *TFCP2L1* | No |  |
| *THRA* | No |  |
| *THRB* | No |  |
| *TLE1* | No |  |
| *TOX3* | No |  |
| *TP63* | No |  |
| *TSHZ1* | No |  |
| *TSHZ2* | No |  |
| *TSHZ3* | No |  |
| *ZBTB20* | No |  |
| *ZBTB7C* | No |  |
| *ZEB1* | No |  |
| *ZEB2* | Yes | Diez-Roux, G., Banfi, S., Sultan, M., et al., 2011. A high-resolution anatomical atlas of the transcriptome in the mouse embryo. PLoS Biol 9, e1000582. |
| *ZFHX4* | No |  |
| *ZFP36L1* | No |  |
| *ZFPM2* | Yes | Ackerman, K.G., Herron, B.J., Vargas, S.O., Huang, H., Tevosian, S.G., Kochilas, L., Rao, C., Pober, B.R., Babiuk, R.P., Epstein, J.A., Greer, J.J., Beier, D.R., 2005. Fog2 is required for normal diaphragm and lung development in mice and humans. PLoS genetics 1, 58-65. |
| *ZMAT4* | No |  |
| *ZNF230* | No |  |
| *ZNF25* | No |  |
| *ZNF302* | No |  |
| *ZNF324B* | No |  |
| *ZNF383* | No |  |
| *ZNF385B* | No |  |
| *ZNF467* | No |  |
| *ZNF521* | No |  |
| *ZNF536* | No |  |
| *ZNF579* | No |  |
